# DETECT I & DETECT II: a study protocol for a prospective multicentre observational study to validate the UroMark assay for the detection of bladder cancer from urinary cells

**DOI:** 10.1186/s12885-017-3758-7

**Published:** 2017-11-15

**Authors:** Wei Shen Tan, Andrew Feber, Liqin Dong, Rachael Sarpong, Sheida Rezaee, Simon Rodney, Pramit Khetrapal, Patricia de Winter, Frelyn Ocampo, Rumana Jalil, Norman R. Williams, Chris Brew-Graves, John D. Kelly

**Affiliations:** 1 Division of Surgery and Interventional Science, University College London, 74 Huntley Street, London, WC1E 6AU UK; 2 Department of Urology, University College London Hospitals, London, UK; 3 UCL Cancer Institute, University College London, London, UK; 4 Surgical & Interventional Trials Unit, Division of Surgery and Interventional Sciences, Faculty of Medical Sciences, University College London, London, UK

**Keywords:** Bladder cancer, Clinical trial, Diagnostic, Haematuria, Methylation, Next generation sequencing, Urinary assay, Urinary biomarker, Surveillance, Validation

## Abstract

**Background:**

Haematuria is a common finding in general practice which requires visual inspection of the bladder by cystoscopy as well as upper tract imaging. In addition, patients with non-muscle invasive bladder cancer (NMIBC) often require surveillance cystoscopy as often as three monthly depending on disease risk. However, cystoscopy is an invasive procedure which is uncomfortable, requires hospital attendance and is associated with a risk of urinary tract infection. We have developed the UroMark assay, which can detect 150 methylation specific alteration specific to bladder cancer using DNA from urinary sediment cells.

**Methods:**

DETECT I and DETECT II are two multi-centre prospective observational studies designed to conduct a robust validation of the UroMark assay. DETECT I will recruit patients having diagnostic investigations for haematuria to determine the negative predictive value of the UroMark to rule out the presence of bladder cancer. DETECT II will recruit patients with new or recurrent bladder cancer to determine the sensitivity of the UroMark in detecting low, intermediate and high grade bladder cancer. NMIBC patients in DETECT II will be followed up with three monthly urine sample collection for 24 months while having surveillance cystoscopy. DETECT II will include a qualitative analysis of semi-structured interviews to explore patients’ experience of being diagnosed with bladder cancer and having cystoscopy and a urinary test for bladder cancer surveillance. Results of the UroMark will be compared to cystoscopy findings and histopathological results in patients with bladder cancer.

**Discussion:**

A sensitive and specific urinary biomarker will revolutionise the haematuria diagnostic pathway and surveillance strategies for NMIBC patients. None of the six approved US Food and Drug Administration urinary test are recommended as a standalone test. The UroMark assay is based on next generation sequencing technology which interrogates 150 loci and represents a step change compared to other biomarker panels. This enhances the sensitivity of the test and by using a random forest classifier approach, where the UroMark results are derived from a cut off generated from known outcomes of previous samples, addresses many shortcomings of previous assays.

**Trial registration:**

Both trails are registered on clinicaltrials.gov. DETECT I: NCT02676180 (18th December 2015). DETECT II: NCT02781428 (11th May 2016).

## Background

Haematuria is a common finding in general practice [1] and requires visual inspection of the bladder by cystoscopy as well as upper tract imaging [2]. Both non-visible and visible haematuria are associated with the presence of bladder cancer [3]. However, bladder cancer is responsible for only 12.0% and 5.2% of visible and non-visible haematuria respectively [4]. Cystoscopy is an invasive procedure which is uncomfortable, requires hospital attendance and is associated with a 5% risk of urinary tract infection [5]. It is estimated that using cystoscopy to investigate haematuria cost the UK healthcare £55 million per year, ranking bladder cancer as one of the most expensive cancers to manage [6]. Furthermore, patients with known non-muscle invasive bladder cancer (NMIBC) will require surveillance cystoscopy as frequently as 3 monthly in high risk cases due to the risk of disease recurrence and progression of between 31%–78% and 17%–45% respectively within 5 years [7]. Hence, a non-invasive urinary test which can rule out the presence of bladder cancer would be of great value.

Two Health Technology Assessment (HTA) commissioned systematic reviews have highlighted the clinical need for urinary based markers for the detection of bladder cancer [8, 9]. Currently available Food and Drug Administration (FDA) approved urinary based markers have failed to become standard practice as they miss a significant number of patients with bladder cancer and are subject to a high number of false positives [10]. Hence, no urinary biomarker has been approved as a standalone test for the detection of bladder cancer and cystoscopy is still recommended.

Epigenetic alterations such as DNA methylation make an ideal non-invasive biomarker for the detection and surveillance of disease given its ontogenic plasticity and tissue specificity. A number of emerging assays based on epigenomic panels have shown the potential utility of DNA methylation changes as urinary biomarkers for the detection of bladder cancer [11, 12]. We have developed the UroMark assay, which can detect 150 methylated specific alteration using DNA from urinary sediment cells. Full details of the development of this assay has been described previously [13]. Unlike previous reported tests, the UroMark assay utilises microdroplet-based polymerase chain reaction (PCR) application followed by next-generation sequencing of amplification of target loci using RainDance Technology, which allows sensitive, specific and simultaneous amplification of up to 20,000 methylated loci [14–16]. DETECT I and DETECT II are two multi-centre prospective observational studies designed to conduct a robust validation of the UroMark assay.

## Methods/design

Both DETECT I and DETECT II are prospective multicentre observational study designed to assess the diagnostic performance of the UroMark. Both studies will recruit patients in >30 centres in the UK.

### Detect I

#### Objectives

The primary objective of DETECT I is to determine the negative predictive value of the UroMark assay to rule out the presence of bladder cancer in patients with haematuria. The secondary objectives include 1) to assess the feasibility of a large scale home urine collection system for high throughput analysis, 2) exploratory health economics analysis of patients’ views on the use of a urine based test and 3) to assess the negative predictive value and positive predictive value of the combination of standard of care imaging with the UroMark in the entire cohort and in pre-defined sub-groups of patients.

#### Study design

The study will recruit patients who are referred for haematuria investigations by their general practitioner (GP). Inclusion criteria include 1) patients ≥18 years of age, 2) undergoing cystoscopy for visible or non-visible haematuria, 3) had upper tract imaging (either ultrasound kidney, ureters and bladder (KUB), CT KUB or CT intravenous urogram (IVU)) within 12 weeks of registration into the study and 4) patients who are able to provide informed consent. Patients who are unwilling to have cystoscopy and upper tract imaging or unable to give informed consent will be excluded.

**Fig. 1:**
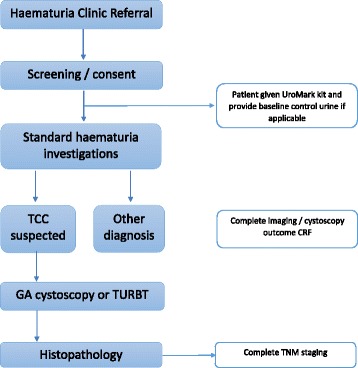
Study schedule for DETECT I

The study schedule for DETECT I is reported in Fig. 1. Written informed consent will be obtained and patients will be screened for eligibility and inclusion into the trial. Patients will be given a UroMark home urine collection kit and samples are mailed to the laboratory in a prepaid postage envelope in packaging specifically designed for this purpose, and approved by Royal Mail. Patients will have standard haematuria investigations which consist of flexible cystoscopy and upper tract imaging. They will also be asked to complete a health economics questionnaire which is designed to capture their opinion and perspectives on their confidence in having a specialised assay to diagnose their bladder cancer instead of cystoscopy. Patients with cystoscopic evidence of tumour will be treated with transurethral resection of bladder tumour (TURBT) as part of standard practice and histopathological evidence of tumour will be used a standard reference. All urine samples are collected prior to TURBT.

The study protocol was approved by Health Research Authority: North West Liverpool Central Research Ethics Committee on the 9 ^th^ March 2016 (IRAS project ID: 179,245, REC reference: 16/NW/0150). DETECT I is registered on clinicaltrials.gov NCT02676180.

#### Assessment

Patient demographics including age, gender, occupation, ethnicity and smoking history will be recorded. Results of flexible cystoscopy, upper tract and bladder imaging and histopathological results (if TURBT or bladder biopsy is performed) will be recorded in an electronic case report form (eCRF).

#### Sample size and power calculations

The primary outcome of DETECT I is the negative predicted value (NPV) of the UroMark assay which is expected to be 98% (or higher). Using the exact binomial method, to give a lower bound for a 95% confidence interval of 96.75%, 800 negative test results are required. It is expected that 90% of all tests would be negative thus; we will require at least 889 evaluable urine samples. With this sample size, the uncertainty in the estimated NPV will be less than 1% if the NPV is higher than 98%. The data analysis will involve calculating the NPV from the data, along with an exact binomial confidence interval. To ensure confidence subjects will be recruited until at least 89 tumours have been detected. It is proposed that the NPV will be examined after a third of cases have been recruited (approximately 300 cases). If the NPV is substantially below that assumed, the study may be terminated early.

For the secondary outcome, the NPV of imaging (ultrasound KUB/CT) alone and the combination of imaging and the UroMark assay will be determined. Specifically, the NPV of UroMark will be calculated for the following subgroups: haematuria type (visible vs non-visible), gender (male vs female), age (≤50 vs >50 years) and smoking history (yes vs no). Fisher’s exact test will be used to determine the NPV.

### DETECT II

#### Objectives

The primary objective of DETECT II is to determine the sensitivity of the UroMark to detect new and recurrent low, intermediate and high grade bladder cancer. Secondary objectives include 1) to determine whether the UroMark can detect NMIBC recurrence in patients undergoing cystoscopic surveillance for bladder cancer, 2) to assess patients’ perspectives on cystoscopy and using a non-invasive urinary test and 3) to use semi-structured patient interviews to qualitatively explore patients experience of being diagnosed with bladder cancer and having cystoscopy and a non-invasive urinary test as a method of bladder cancer surveillance.

#### Study design

The study will recruit patients with new or recurrent bladder cancer. Patients who are not willing to have TURBT or unable to give consent will be excluded.

**Fig. 2:**
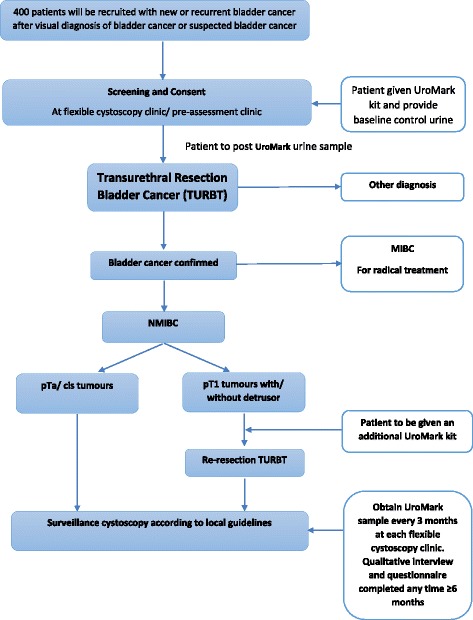
Study Schedule DETECT II

The DETECT II study schedule is reported in Fig. 2. Patients will be screened and included into the trial at flexible cystoscopy after visual confirmation of new or recurrent bladder tumour. Written consent will then be obtained from eligible patients. A baseline urine sample will be collected using the same home urine collection kit being used in DETECT I. Urine collection must be performed prior to TURBT. Histopathological confirmation of tumour stage (Ta, T1, ≥T2) and grade (G1, G2, G3) with or without CIS, or isolated CIS will be used a reference standard.

All patients will receive standard test and investigations for bladder cancer. Following a TURBT, it is recommended that patients with NMIBC will receive a single instillation of intravesical chemotherapy unless clinically contraindicated. Intermediate risk NMIBC will have an inductive course of intravesical chemotherapy post TURBT while high risk NMIBC may require a repeat TURBT, usually within 6 weeks of the first TURBT. The repeat resection is recommended for high risk tumours to exclude residual detrusor muscle invasion (pT2 at least). High risk patients will either have an inductive and maintenance course of intravesical BCG or a radical cystectomy. This is consistent with international consensus [17].

Patients with NMIBC will have periodic cystoscopic surveillance following TURBT between 3 to 12 month intervals depending on disease risk in accordance to local hospital guidelines. Patients will be asked to provide a urine sample using a UroMark urinary collection kit every 3 months for 24 months (Table 1). It is important that urine samples are collected prior to TURBT to capture urine positive for recurrent tumour.

**Table 1:** Table of Assessments in DETECT II

Visit	Baseline Before TURBT	3 Month	6 Month	9 Month	12 Month	15 Month	18 Month	21 Month	24 Month
Inclusion criteria	X								
Smoking history	X								
Visual Diagnosis of bladder cancer	X								
Surveillance Cystoscopy:									
Low risk		X			X				
Intermediate risk		X		X			X		
High risk		X	X	X	X	X	X	X	X
UroMark Test	X	X	X	X	X	X	X	X	X

NMIBC patients undergoing surveillance will complete a questionnaire designed to assess their perspectives on cystoscopy compared with a urinary test as part of surveillance to detect bladder cancer recurrence (Table 1). Selected patients will be invited for a telephone interview to further explore their experience of being diagnosed with bladder cancer and having cystoscopy and a urinary test as a method of bladder cancer surveillance. Up to 40 patients will participate in a qualitative analysis of semi-structured interviews but this number may be lower if patient responses have reached a saturation point. Patients will be interviewed after a minimum of 6 months’ follow-up to ensure all patients will be able to provide an informed opinion after having both diagnostic investigations: cystoscopy and urine based test.

DETECT II study protocol received Health Research Authority: London- Stanmore Research Ethics Committee approval on the 30th August 2016 (IRAS project ID: 203,022, REC reference: 16/LO/1044). This trial is registered on clinicaltrials.gov NCT02781428.

#### Assessment

Patient demographics including age, and smoking history will be recorded as well as cystoscopy and TURBT or bladder biopsy histopathology results. Any adjuvant treatment post endoscopic management will also be recorded. Registration of patients and recording of all data is done electronically. However, results of the UroMark will not be reported to the treating clinicians to ensure there is no deviation from standard of care. Results of each surveillance cystoscopy will be recorded and any subsequent management. All results will be recorded in an online eCRF.

#### Sample size and power calculations

The primary aim of DETECT II is to determine the sensitivity of the UroMark assay in detecting Grade 1, 2 and 3 bladder cancers. It is proposed that at least 380 urine samples will be required from bladder cancer patients. This sample size is based on the assumption of 95% sensitivity and a 95% confidence interval ranging between 92.3% and 97.0%. It is essential to test the ability of the UroMark assay to detect low grade bladder cancer and these cancers will represent at least 15%–20% of disease detected. In order to reduce the uncertainty in estimating sensitivity, we aim to enrich the cases recruited with at least 100 cases from other clinical trials (including 60 with low grade disease). It is estimated that the sensitivity for detection of these cancers will be 80% which will provide a 95% CI of between 70.8% to 87.3%.

## Discussion

There are six FDA approved urinary biomarkers which are available commercially for clinical use. These are: BTA stat (Polymedco), BTA TRAK (Polymedco), NMP22 BC test kit (Matritech), NMP22 BladderCheck Test (Alere), ImmunoCyt (Scimedx) and UroVysion Bladder Cancer Kit (Abbott Molecular). These biomarkers have a sensitivity between 57 and 82% and a specificity of 74–88% [10] and in all tests, the sensitivity was higher in high grade and stage tumours. Bladder cancer is a heterogeneous disease and an inherent flaw of currently available commercial assays are the reliance on single or small panel of markers, and none are licensed to be used without cystoscopy [18]. For example, the UroVysion uses 5 genomic probes and NMP22 Bladdercheck detects a single protein.

Studies on methylation specific markers have reported a sensitivity of between 65 and 100% and specificity of 77–100% [19]. The majority of these studies use methylation specific PCR to profile only a few genes (between one to six) due to the limited amount of DNA which can be extracted from urinary sediment [20]. However, none of these novel assays have been regulatory approved. Given that bladder cancer is a heterogenous disease, it is possible that more genes would be necessary to detect the presence of bladder cancer from urinary DNA [21].

The UroMark assay is novel in concept and designed to interrogate 150 methylation specific loci which will enhance test sensitivity. The number of markers used previously has been limited by the amount of DNA from urinary cell pellet. However, using RainDance Microdroplet PCR as previously described, this limitation has been overcome [14, 15]. In addition, the UroMark assay uses a random forest classifier which analyses the methylation status for each of 150 loci [22]. The classifier does not rely on single or low number of positive markers, or, a predefined pattern of methylation across a set of markers and a dichotomous output is derived from a cut off generated from the known outcomes of prior samples. To our knowledge this the first approach using high throughput sequencing technology for the development of a bladder cancer detection assay and it addresses many of the shortcomings of previously assays.

Most studies reported previously were retrospective studies comparing results of a urinary biomarker in a cohort of bladder cancer patients with controls. In DETECT I, all patients recruited are referred by their GP for haematuria evaluation. These patients are typically investigated by cystoscopic evaluation of the bladder and an upper tract scan within two weeks in dedicated haematuria clinics in urology departments throughout the UK. It is estimated that there are over 100,000 haematuria referrals by GPs in the UK [23]. Similar to DETECT I, recruitment into DETECT II should be achievable given there are >12,000 new bladder cancer cases per year in England and Wales [24]. Furthermore, using prospectively collected urine samples from bladder cancer trials in low and intermediate risk bladder cancer patients will allow us to enrich for this group of patients which has proved challenging for other non-invasive urine tests.

DETECT I will also test the feasibility of changing the haematuria diagnostic pathway from one which requires a hospital visit to a system where potentially GPs can request a bladder cancer diagnostic investigations without the need for the patient to attend a hospital clinic. DETECT II will provide data to determine if periodic urine testing of NMIBC patients for DNA methylation changes to interrogate recurrence would be as effective as cystoscopy. This is a novel approach, and a convenient and cost-effective alternative. Reporting of patients’ views of using a urine based test compared to cystoscopy has been limited [25, 26]. A qualitative analysis of semi-structured interviews in a cohort of NMIBC patients having both surveillance cystoscopy and using a urinary test will allow an assessment of patients’ views and interrogate the complexities and subtleties of their decision making process which has not been performed previously.

## Conclusion

DETECT I and II is a multi-centre prospective observational studies designed to conduct a robust validation of the UroMark assay. DETECT I will validate the ability of the UroMark assay to rule out bladder cancer in patients with haematuria while DETECT II will determine the diagnostic accuracy of the assay in a patient cohort enriched for low grade cancer and test the ability of the UroMark assay to detect bladder cancer recurrence. We aim to show sufficient diagnostic precision to replace cystoscopy in the evaluation of patients with haematuria. The incorporation of a highly sensitive and specific assay will revolutionise bladder cancer pathways, have a profound impact on the requirement for cystoscopy and on patient well-being and, reduce the healthcare costs associated with investigation of haematuria and surveillance for disease recurrence.

## Trial status

DETECT I commenced recruitment on the 30th March 2016 and anticipated to close on 1st Feb 2019. DETECT I was approved by the Health Research Authority: North West Liverpool Central Research Ethics Committee on the 9th March 2016. DETECT II commenced recruitment on the 26th September 2016 and will likely close on 25th September 2019. DETECT II was approved by the Health Research Authority: London Stanmore Research Ethics Committee on the 30th August 2016. Both trials are currently recruiting patients and are registered on clinicaltrials.gov.

## Abbreviations

BTA: Bladder tumour antigen
CT KUB: Computer tomography of the kidneys, ureters and bladder
DNA: Deoxyribonucleic acid
eCRF: electronic case report form
FDA: Food and Drug Administration
FISH: Fluorescence in situ hybridization
HTA: health technology assessment
IVU: Intravenous urogram
KUB: Kidney, ureters and bladder
NMIBC: non-muscle invasive bladder cancer
NMP22: nuclear matrix protein 22
NPV: Negative predictive value
PCR: polymerase chain reaction
TURBT: Transurethral resection of bladder tumour
